# Validated chromatographic methods for determination of teriflunomide and investigation of its intrinsic stability

**DOI:** 10.1186/s13065-024-01186-3

**Published:** 2024-05-03

**Authors:** Shereen A. Boltia, Mai M. Mora, Nahla S. Ismail, Hala E. Zaazaa

**Affiliations:** 1 Analytical Chemistry Department, Faculty of Pharmacy, Cairo University, Kasr El-Aini St, Cairo, 11562 Egypt; 2 Egyptian Drug Authority (EDA), Giza, Egypt

**Keywords:** RP-HPLC, Stability indicating, Teriflunomide, HPTLC, Validation

## Abstract

Two rapid, precise, and sensitive stability-indicating high performance chromatographic methods for the measurement of Teriflunomide in its degradation products’ existence were developed. These were RP-HPLC and HPTLC using UV detector. HPLC separation was accomplished utilizing Thermo BDS hypercil C18 column (250 × 4.6 mm, 5 μm) and acetonitrile: 0.03 M potassium dihydrogen phosphate: triethylamine (50:50:0.1%, by volume) as mobile phase at flow rate of 1mL/min. The separated peaks were detected at 250.0 nm. The densitometric approach was conducted utilizing HPTLC 60 F254 silica gel plates, and a developing system of benzene: ethanol: acetic acid (7.5:1:0.25, by volume) and detection was done at 250.0 nm. The developed approaches were evaluated regarding the International Conference on Harmonization (ICH) instructions. The calibration curves of both techniques were constructed with linearity ranges of (5-100) µg/mL and (2–10) µg /band, for HPLC and densitometric determination, consecutively. Teriflunomide was exposed to base and acid hydrolysis, oxidation using H_2_O_2_ and finally, thermal degradation as stated in ICH guidelines. The degradation product structures’ elucidation was achieved through LC-MS.

## Introduction

**Fig. 1:**
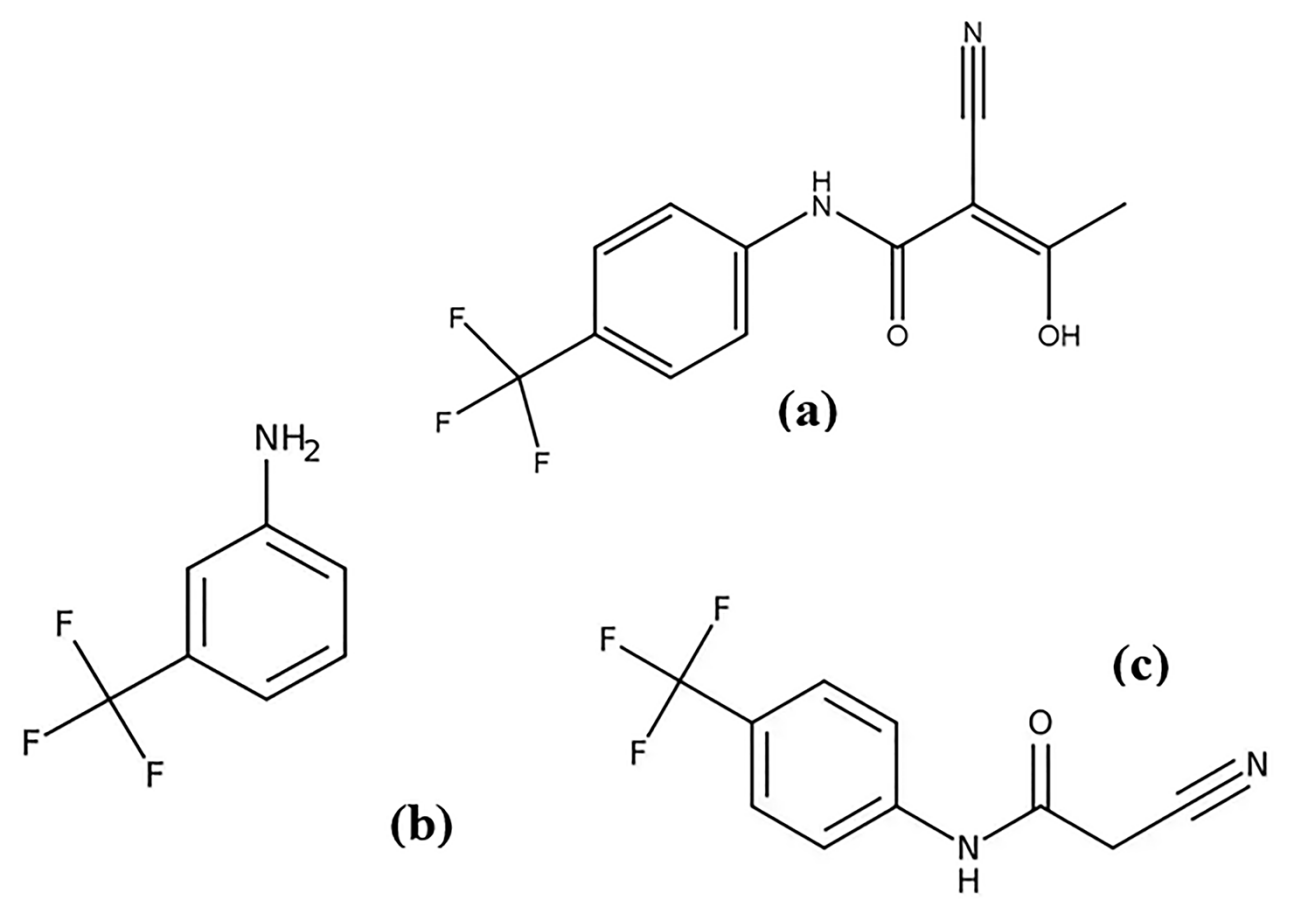
Chemical Structures of: **(a)** Teriflunomide (TER). **(b)** 4-(trifIuoromethyl) aniline (IMPA). **(c)** 2-cyano-N-[4-(trifIuoromethyl) phenyl] acetamide (IMP B)

Teriflunomide (TER) is an immunosuppressive agent and the primary active metabolite of leflunomide. It is used to prevent organ and tissue transplant rejection, such as in heart, bone marrow, kidney, and liver transplantation, as well as for the treatment of autoimmune disorders including rheumatoid arthritis, Behcet’s Disease, multiple sclerosis, ulcerative colitis, and pemphigus [1]. Teriflunomide acts by suppressing the production of new pyrimidines through the inhibition of the enzyme dihydro-orotate dehydrogenase [2–5]. The drug was approved by the FDA on September 13, 2012, and by the European Union on August 26, 2013. Teriflunomide, depicted in Fig. (1a), has the chemical formula C_12_H_9_F_3_N_2_O_2_ and a molecular weight of 270.21 g/mol [6].

A review of the literature revealed that only a limited number of methods, including HPLC [7, 8], HPTLC [9], and LC-MS/MS [10–14], have been reported for the measurement of TER, but without the identification or elucidation of TER degradation products. Two compounds, 4-(trifluoromethyl) aniline with a molecular weight of 161.12 g/mol (Fig. 1b), and 2-cyano- N-(4-trifluoromethylphenyl) acetamide with a molecular weight of 228.17 g/mol (Fig. 1c), are listed in the British Pharmacopoeia (BP) as impurity A (IMP A) and impurity B (IMP B) of TER, respectively.

The primary objective of our study is to develop, optimize, and validate stability-indicating methods that can accurately estimate TER in the presence of its degradation products. These methods should be capable of differentiating and quantifying TER from the impurities, ensuring the reliability and specificity of the analysis.

Therefore, the aim of the proposed chromatographic techniques was to develop and evaluate robust, rapid, sensitive, and precise stability-indicating HPLC and HPTLC methods for the analysis of TER in bulk powder and pharmaceutical dosage forms, considering the presence of its degradation products. Additionally, LC-MS technique was used to confirm that the aforementioned IMP A and IMP B are the resultant compounds after complete degradation of TER, thus validating this assumption.

## Experimental

### Instrumentation

#### HPLC

HPLC system (Agilent series 1200, USA), prepared with a quaternary pump, thermostatic Auto-Sampler and UV detector. Isolation and quantitation were made utilizing Thermo BDS hypersil (250 × 4.6 mm, 5 μm particle size) column. Data gathering, data management, and instrumentation control were conducted by open LAB version and sonicator (Crest, USA) was used.

#### TLC-densitometric method

The Camag Linomat 5 autosampler and Camag micro syringe were employed (CAMAG, Muttenz, Switzerland). Camag TLC scanner 3 model 3 densitometer outfitted with WINCATS software for densitometric assessment, a short wavelength UV lamp producing at 250.0 nm (VILBER LOURMAT, France), slit dimension: 60.3 mm; scanning speed: 20 mm/s; data resolution: 100 m/step; bandwidth: 3 mm. Thin-layer chromatographic plates with high performance, pre-coated with silica gel 60 F254, 20 × 20 cm, 0.25 mm thickness, Merck, Darmstadt.

#### LC-MS

Elucidation of TER degradation products was performed by triple quadrupole mass spectrometer (model 6420) (USA).

### Material and reagents

#### Pure standard

Pure Teriflunomide standard and 2-cyano- N-(4-trifluoromethylphenyl) acetamide were kindly supplied by Sanofi-Aventis Deutschland GmbH both with purity 99.9% according to certificate of analysis and 4-(trifluoromethyl) aniline was supplied from Sigma Aldrich.

#### Pharmaceutical formulation

Aubagio® film coated tablets (B.N.8T22p), synthesized by (Sanofi-Aventis-France) labelled to contain14 mg Teriflunomide was gathered from local market.

#### Chemicals and reagents

- Chemicals of analytical grade were potassium dihydrogen phosphate, triethylamine, acetic acid and benzene (Scharlab, Spain).
- Hydrogen peroxide solution, 30% (w/w) in H_2_O, sodium hydroxide and hydrochloric acid (Sigma Aldrich) used for degradation.
- Solvents of HPLC grade were; acetonitrile, ethanol and methanol (Fisher Scientific, UK).
- Double distilled deionized water (Otsuka, Cairo, Egypt).

### Preparation of standard solution

#### RP-HPLC procedure

##### Stock standard solutions

Stock standard solution (100 µg/mL) of TER was made by transferring accurately weighed10 mg of TER bulk powder into 100-mL volumetric flask and volume was -made with the diluent, (acetonitrile: water) 50:50, v/v. the prepared solution was sonicated for 3 min till complete dissolution.

##### Working standard solution

Working standard solution (30 µg/mL) was generated by transferring 3.0 mL of TER stock standard solution (100 µg/mL) into a 10-mL volumetric flask and then diluting to volume with the same solvent.

#### HPTLC Method

##### Stock standard solutions

Ten mg of TER were accurately weighed and transferred into 100-mL volumetric flask. The volume was up to the mark with methanol to reach final concentrations of100 µg/mL.

##### Working standard solution

Five mL were transferred from 100 µg/mL stock solution into 10 mL volumetric flask and volume was reached by the same dilutant to give a final concentration of 50 µg/mL of TER.

### Preparation of Stock Solution of Dosage Form

#### RP-HPLC method

Ten Aubagio® tablets, each containing 14 mg of TER, were weighed and crushed using a pestle and mortar. The resulting powder weighing 10 mg of TER was transferred into a 100 mL volumetric flask after precise weighing. To this flask, 50 mL of diluent (acetonitrile: water, 50:50 v/v) was added, followed by sonication for 5 min. The volume was then completed, resulting in the preparation of a TER stock solution with a concentration of 100 µg/mL.

#### HPTLC Method

The test was prepared by the same way as under 2.4.1. RP-HPLC but after using methanol instead of diluent.

### Forced degradation studies

The optimum degradation was achieved after several trials of changing concentration of HCl, NaOH, H_2_O_2_ and temperature. The following conditions give the best results of degradation.

#### Acid and base degradation

A total of 10 mg of TER was weighed and placed into a 100 mL flask. For acid hydrolysis, 10 mL of 2 N HCl was added, while for base hydrolysis, 10 mL of 2 N NaOH was added. The flask was then refluxed for 2 h. After the hydrolysis process, neutralization was achieved by adding the precalculated amount of 2 N NaOH (for acid hydrolysis) or 2 N HCl (for base hydrolysis). The resulting solution was filtered using a 0.45µ Polyvinylidene difluoride (PVDF) membrane filter. Finally, the volume was adjusted to 100 mL with the diluent.

#### Oxidative degradation

Ten milligrams of TER were weighed and placed into a 100 mL flask. Then, 10 mL of 30%.

H_2_O_2_ (hydrogen peroxide) were added to the flask, and the mixture was refluxed for 2 h. After the refluxing period, the solution was evaporated to dryness to remove any remaining oxygen. The residue was then reconstituted in 100 mL of diluent. Next, the solution was filtered using a 0.45µ PVDF membrane filter, and the volume was adjusted to 100 mL using the diluent.

#### Thermal degradation

Ten milligrams of TER were weighed and placed into a 100 mL flask. Then, 10 mL of diluent were added, and the solution was heated at 80 °C for two hours. After the heating period, the solution was filtered using a 0.45µ PVDF membrane filter, and the volume was adjusted to 100 mL using the diluent.

During all the previous processes, a blank solution was prepared without the addition of the sample. Both the blank and sample solutions were introduced into the HPLC system for analysis.

### Procedure

#### RP-HPLC technique

##### Chromatographic circumstances

The separation was carried out using an Agilent 1200 Series system with an RP-C18 Thermo BDS hypersill column (please refer to the Instrumentation section for HPLC details). Isocratic elution was performed using a mobile phase consisting of acetonitrile, 0.03 M potassium dihydrogen phosphate, and triethylamine in a ratio of 50:50:0.1% (by volume). Prior to use, the mobile phase components were filtered through a 0.45 mm Millipore membrane filter and vented in situ for 15 min in an ultrasonic bath. The mobile phase was delivered at a flow rate of 1.0 mL/min. Before injection, the samples were filtered through a 0.45 mm membrane filter and injected in 5 µL volumes. The UV detection was performed at 250 nm and at room temperature.

##### Calibration curve construction

Aliquots of TER were accurately transferred from the 100.0 µg/mL stock standard solutions into a series of 10-mL volumetric flasks. The flasks were then filled to the top with diluent, resulting in solutions with a concentration range of 5.0–100.0 µg/mL. These prepared solutions were injected in triplicate into the HPLC system using the previously mentioned chromatographic conditions. To construct a calibration curve, the peak area of each concentration was correlated with the corresponding concentrations in µg/mL. Regression formulas were calculated based on this data.

##### Application to pharmaceutical preparation

Three mL were pipetted out from stock solution previously prepared under ***2.4.1 Preparation of Stock Solution of Dosage Form for RP-HPLC*** then the volume was diluted up to 10 mL with diluent to gather a final concentration of 30 µg/mL of TER.

#### HPTLC approach

##### Chromatographic circumstances

For the HPTLC analysis, the samples were prepared in triplicates as bands on 20 × 10 cm HPTLC plates using a Camag Auto-sampler. The bands were applied 1.0 cm above the bottom edge of the plate and had a length of 3.0 mm. There was a 1.0 cm space left on both the right and left sides of the bands.

The chromatographic chamber was saturated with the developing system, which consisted of benzene, ethanol, and acetic acid in a ratio of 7.5:1:0.25 (by volume), for 30 min at room temperature. After saturation, the plates were placed in the chamber for ascending development, covering a distance of 9.0 cm.

Once the development was complete, the plates were removed from the chamber, allowed to air dry, and then scanned at a wavelength of 250.0 nm.

##### Construction of calibration curve

Precisely measured quantities of TER in the range of 2–10 µg per band were applied as separate bands on the HPTLC plates using the established chromatographic conditions mentioned earlier. This process was repeated three times for each concentration.

After the bands were applied, the scanning profiles of the plates were collected. From these profiles, calibration curves were constructed by plotting the peak area of each band against its corresponding concentration. Polynomial correlations were established between the integrated peak areas and the concentrations of TER. Regression formulas were then calculated based on these correlations.

##### Method validation

Proposed methods’ Validation has been done as per ICH instructions [15].

##### ***Linearity and range***

Six different concentrations of TER were analysed in triplicates by the proposed chromatographic methods. Calibration curves were constructed to relate the peak area to the subsequent concentration of TER.

##### ***Accuracy***

Under these conditions, 9 separate determinations were made across all three concentration levels utilizing the process, and the results were examined to determine the precision of the devised HPLC and HPTLC procedures. We then used the resulting regression models to probe the percentages of losses that were recovered.

##### ***Precision***

Three different concentrations of TER in triplicates (5, 30and 70 *µg*/mL) for HPLC approach and (2, 3, 4*µg*/band) for HPTLC were analysed within a day (repeatability) and in three various days (intermediate precision). The proportion of relative standard deviation (RSD %) was computed for both methods.

##### ***Detection and quantitation limits***

LOQ and LOD were computed using residuals standard deviation and slope of each method, as shown in Table 2. The detection limit (DL) can be expressed as: 𝐷𝐿 = 3.3𝜎 / S while the quantitation limit (QL) can be expressed as: 𝑄𝐿 = 10𝜎 /𝑆, where σ = the standard deviation of the response S = the slope of the calibration curve and the estimate of σ based on the standard deviation of the blank.

**Table 1:** System Suitability Parameters for the recommended HPLC and HPTLC methods for the teriflunomide’s determination

Parameter	Obtained value		Acceptance values
	HPLC	HPTLC	
Retention time (t_R_) (min.)	3.2	0.43	
Tailing factor (T)	1.12	1.5	1 for a typical symmetric peak
Column efficiency (N)(plates/column)	4500	-----	Increase with efficiency of separation.
Resolution (Rs)	7	2	Rs > 1
Selectivity factor (α)	2.5	1.5	As α increases, better resolution is obtained
Capacity factor(K´)	1	2	1–10.

a. Height equivalent to theoretical plate (cm/plate)

b. α = k‘_2_/k‘_1_,where k‘ is the capacity factor

c. Rs= [2(Rf_2_-Rf_1_)]/W_1_ + W_2_), where ‘Rf’ is retardation factor and ‘W’ is peak width

**Table 2:** Suggested HPLC and HPTLC techniques’ regression and validation variables for teriflunomide’s determination in pure powder forms

Parameters	HPLC	HPTLC
**Linearity Range**	5-100µg/mL	2–10 µg/band
**Slope**	33.75	88.99
**Intercept**	12.2	785.2
**Correlation Coefficient**	0.9999	0.9998
**Accuracy (mean ± SD)**	100.51 ± 0.41%	101.01 ± 0.32%
**Precision**:		
**Repeatability** ^**(a)**^	0.751	1.252
**Intermediate precision** ^**(b)**^	1.245	1.855
**LOD**	1µg/mL	0.5 µg/band
**LOQ**	3µg/mL	1 µg/band
**Robustness(pooled)**	0.980	1.606

^(a)^The intraday precision(*n* = 9) of determination of (5, 30 and 70 µg/mL) for HPLC approach and (2, 3, 4 µg/band) for HPTLC were analyzed triplicate within a day

^(b)^The interday precision (*n* = 9) of determination of (5, 30 and 70 µg/mL) for HPLC approach and (2, 3, 4 µg/band) for HPTLC were analysed triplicate in three different days

##### ***Robustness***

Robustness, in the context of analytical chemistry and method validation, refers to the ability of an analytical method to remain unaffected by small, deliberate variations in experimental conditions. It assesses the method’s stability and reliability when slight changes are introduced to factors such as equipment, environmental conditions, or operator techniques. Robustness testing is an essential part of method validation, helping to ensure that the method can provide accurate and consistent results under different circumstances. Different factors were studied for both methods including; variation in wavelength buffer concentration and mobile phase ratio.

##### Application to pharmaceutical preparation

Five milliliters of the stock solution prepared according to Sect. 2.4.2 *(Preparation of Stock Solution of Dosage for RP-HPTLC)* were pipetted out. This volume was then diluted with methanol up to a final volume of 10 mL, resulting in a concentration of 50 µg/mL of TER.

From this diluted solution, a concentration of 4 µg per band was applied on the HPTLC plates.

## Results and discussion

Pharmaceutical products and active pharmaceutical ingredients undergo stability-indicating tests to monitor their degradation and changes in the concentration of the active pharmaceutical ingredients (APIs). These tests are crucial for ensuring the quality and safety of pharmaceutical products. The International Council on Harmonization of Technical Requirements for Pharmaceuticals for Human Use (ICH) has provided recommendations, such as Q3B, Impurities in New Drug Products, which outline the requirements for analyzing degradation products and impurities. These recommendations emphasize the need for validated analysis techniques that are reliable, specific, and capable of detecting and quantifying impurities and degradation products. To meet these requirements, stability-indicating assays utilize various methods. Two commonly employed techniques are high performance thin layer chromatography (HPTLC) and high-performance liquid chromatography (HPLC) [16]. These chromatographic methods offer reliable separation and detection of impurities, ensuring that they are well-separated from the API and other components present in the pharmaceutical formulation. By employing these stability-indicating assays, pharmaceutical manufacturers can effectively monitor and control the quality and stability of their products, ensuring that they meet the regulatory standards set by organizations like the ICH.

The separation and quantitation of the active pharmaceutical ingredient (API), teriflunomide (TER), in the presence of its identified impurities, 4-(trifluoromethyl) aniline (IMP A) and 2-cyano-N-(4-trifluoromethylphenyl) acetamide (IMP B), pose a significant challenge due to their structural similarities and expected similar physical and chemical properties. These impurities are formed as a result of forced degradation processes such as hydrolysis, oxidation, and thermal degradation.

In addition to their presence as degradation products, the teriflunomide metabolites, IMP A and IMP B, have been identified in single and multiple dose toxicology studies. These studies have shown similar toxicity results to those observed with teriflunomide, indicating potential adverse effects associated with these impurities. Increased turnover of red blood cells and oxidative damage have been observed, along with secondary effects such as hemosiderosis and extra-medullary hematopoiesis. High doses of these impurities have also been linked to discolored lungs and livers, suggesting hemorrhages as a possible consequence [17].

Therefore, it is crucial to develop robust stability-indicating methods that can accurately quantify TER in the presence of its degradation products and impurities, enabling the assessment of product quality and safety in pharmaceutical formulations.

### Method optimization

#### HPLC Method

Several experiments were conducted to investigate the factors influencing the chromatographic separation efficiency of TER and its degradation products. Initial trials using different ratios of potassium dihydrogen phosphate and acetonitrile as the mobile phase in an isocratic elution mode provided reasonable results. However, the resolved peaks exhibited some tailing, which negatively impacted the system suitability parameters.

**Fig. 2:**
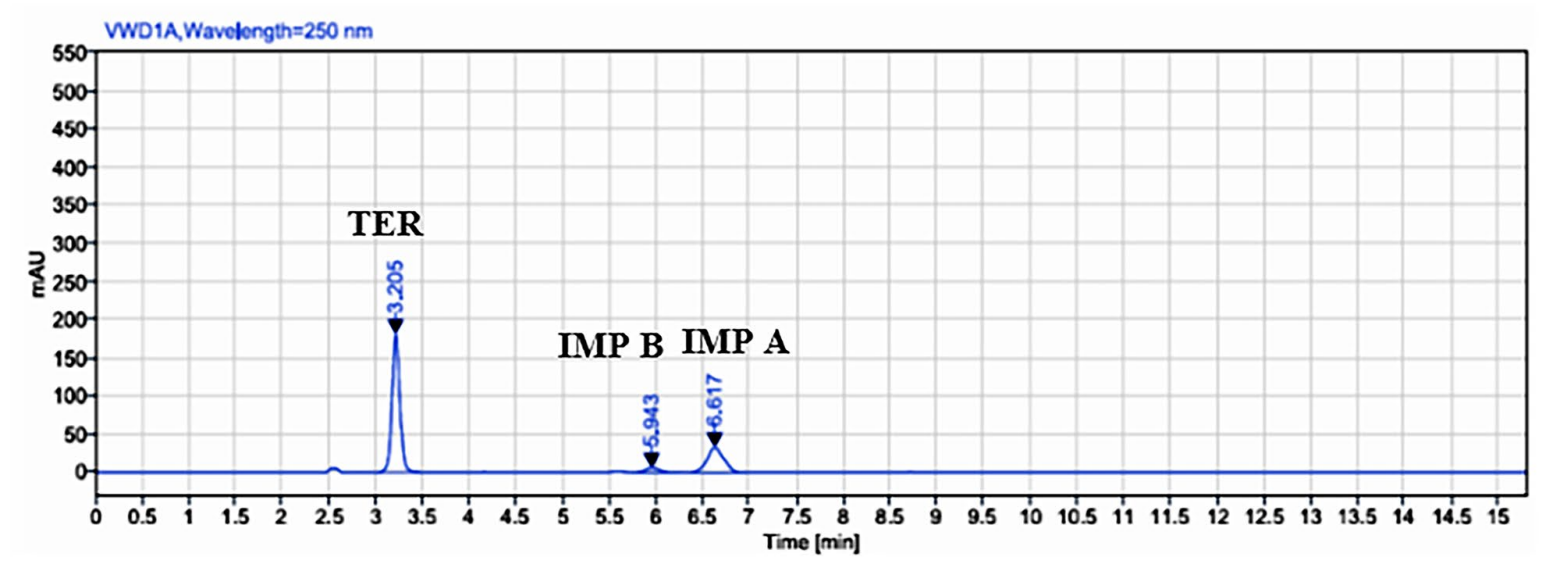
HPLC chromatogram of teriflunamide with its two impurities. **(a)** Teriflunamide (100 µg. mL^-1^). **(b)** 4-(trifIuoromethyl) aniline (50 µg. mL^-1^) IMP A. **(c)** 2-cyano-N-[4-(trifIuoromethyl) phenyl] acetamide (50 µg. mL^-1^) IMP B

To address this issue, triethylamine was added to the mobile phase, leading to improved results, particularly in terms of reducing tailing of the peaks. The optimized chromatographic conditions resulted in good separation, acceptable system suitability parameters, satisfactory resolution, and a suitable analysis time, as depicted in Fig. 2.

System suitability factors were calculated for each approach and compared with the reference values specified in the USP pharmacopeia. The results of the comparison are presented in Table 1, demonstrating that the developed method meets the required system suitability criteria.

Overall, the optimization of the chromatographic conditions and the addition of triethylamine proved to be effective in improving the separation efficiency and achieving desirable system suitability parameters for the analysis of TER and its degradation products.

Linear associations were established between the concentration range of 5.0–100.0 µg/mL and the corresponding peak areas of teriflunomide. The regression equation was calculated to determine the relationship between the concentration and peak area, and the results are presented in Table 2. The limit of quantification (LOQ) and limit of detection (LOD) of teriflunomide were determined from the calibration curve following the guidelines provided by the International Council on Harmonization (ICH), and the values are summarized in Table 2.

To evaluate the robustness of the method, small variations were introduced in specific parameters. These variations included a wavelength change of ± 5 nm (250 ± 5 nm), a buffer concentration change of ± 10% (0.05 M ± 10%), and a ratio change of buffer to acetonitrile of ± 5%. Each parameter was varied while keeping the others constant. The data on the drug’s peak area and tailing factor under these conditions were collected and presented in Tables 3 and 4.

**Table 3:** Robustness results of the proposed HPLC and HPTLC methods for the determination of TER

Method	Parameters	Area	Tailing factor
HPLC	Wavelength (250 ± 5)	0.510	1.211
	Buffer concentration (0.05 M ± 10%)	0.651	1.553
	Ratio of buffer and acetonitrile ± 5%.	0.820	1.139
	**RSD%**	**0.660**	**1.301**
	**Pooled RSD%**	**0.980**	
HPTLC	Wavelength (250 ± 5)	1.103	1.834
	Change in ratio of mobile phase by using 0.3 and 0.2 mL of acetic acid instead of 0.25 mL	1.532	1.956
	**RSD%**	**1.317**	**1.895**
	**Pooled RSD%**	**1.606**	

**Table 4:** Determination of teriflunomide in aubagio® tablets by the suggested HPLC and HPTLC approaches and usage of standard addition technique

Pharmaceutical Product	% Recovery		Standard Addition Technique					
	HPLC	HPTLC	HPLC			HPTLC		
Aubagio® tablets			Added (µg/mL)	Found (µg/mL)	% Recovery	Added (µg/band)	Found (µg/band)	% Recovery
B.N.: 8T22P Claimed to contain 14 mg of TER/tablet	**100.01%±0.22**	**100.55%±0.45**	10.00	10.11	101.00	2.00	1.99	99.50
			30.00	30.51	101.70	3.00	3.06	102.00
			60.00	60.91	101.51	4.00	4.08	102.00
**Mean**					**101.40%±0.36**			**101.16%±0.37**
**SD**					**0.36**			**1.44**
**RSD%**					**0.356**			**1.423**

The results indicate that the reliability and reproducibility of the method were not significantly affected by these small variations in the experimental parameters. This demonstrates the robustness of the developed method, as even minor changes in wavelength, buffer concentration, and ratio of buffer to acetonitrile did not have a substantial impact on the measured peak areas and tailing factors.

#### HPTLC Method

**Fig. 3:**
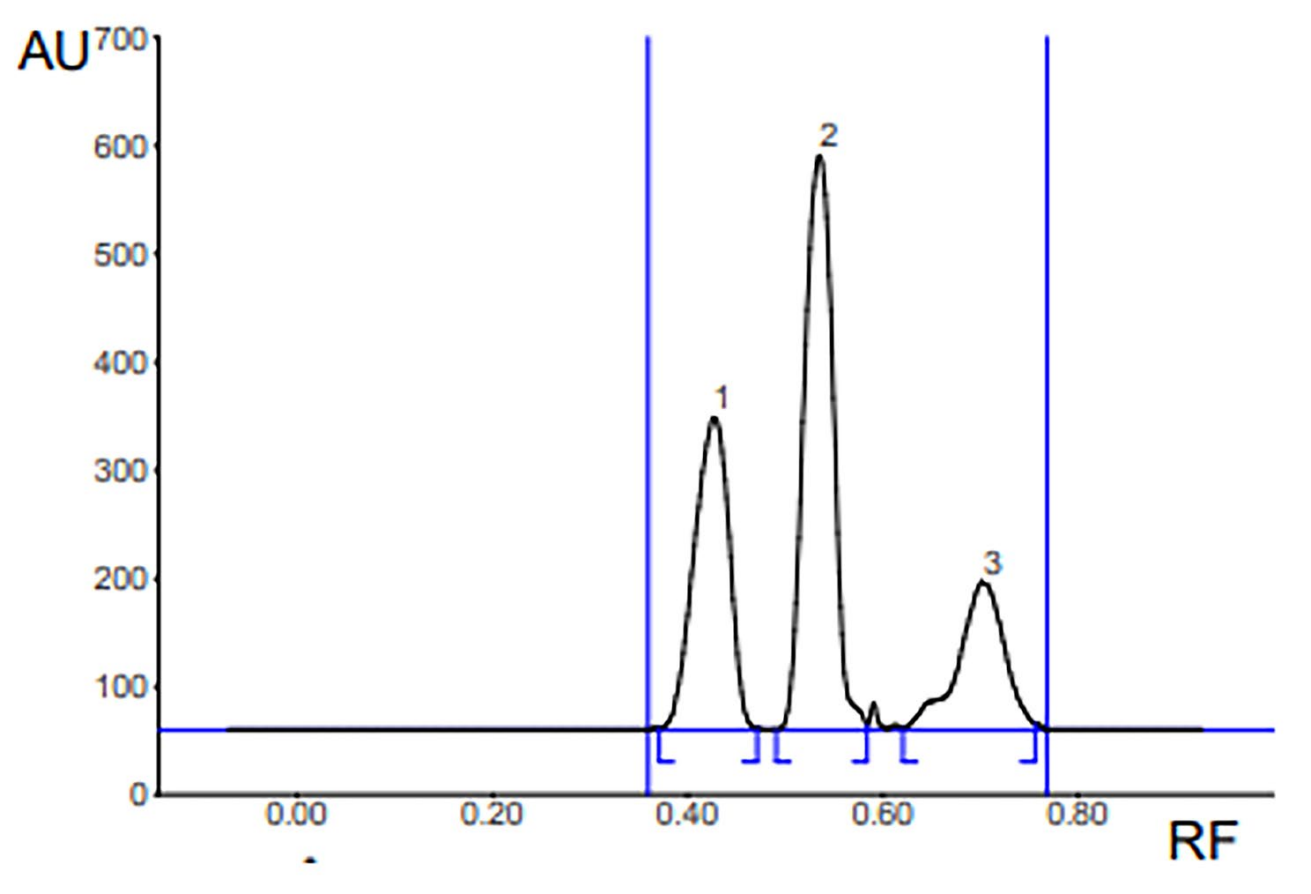
2D HPTLC densitogram at 250 nm of: (1) TER (10 µg. band^-1^), Rf= 0.43. (2) IMP B (10 µg. band^-1^), Rf= 0.54. (3) IMP A (10 µg. band^-1^), Rf= 0.7

The HPTLC-densitometric method was developed and validated through several trials to determine the optimal conditions for the quantitative determination of the studied compound (teriflunomide) in the presence of its impurities. Different developing systems were tested, including methanol: chloroform (8:2, v/v), methanol: hexane (3:7, v/v), ethyl acetate: methanol: water (7:2:1, by volume), and toluene: ethanol (8:2, v/v). However, the best results in terms of complete separation, optimal resolution, and acceptable system suitability parameters were achieved when using benzene: ethanol: acetic acid (7.5:1:0.25, by volume) as the developing system, as shown in Fig. (3).

System suitability factors were calculated for each approach and compared to the reference values specified in the USP pharmacopeia. The results of the system suitability parameters are presented in Table 1, indicating that the developed method using the benzene: ethanol: acetic acid system met the required criteria and exhibited satisfactory performance for the quantitative determination of teriflunomide and its impurities.

The validated HPTLC-densitometric method provides a reliable and robust approach for the analysis of teriflunomide and its impurities, ensuring accurate and precise quantification in pharmaceutical samples. Calibration curves were constructed to establish the relationship between the concentrations in the range of 2–10 µg/band and the corresponding peak areas. Various concentrations of teriflunomide were prepared to determine the linearity of the method, and the limits of quantification (LOQ) and detection (LOD) were calculated based on the standard deviations of intercepts. The proposed method validation was completed by assessing accuracy, linearity, precision, LOQ, and LOD, as summarized in Table 2.

To evaluate the robustness of the method, small variations were introduced in the ratio of the mobile phase by using 0.3 and 0.2 mL of acetic acid instead of the original 0.25 mL, and slight changes in the wavelength (250 ± 5 nm) were examined. While keeping the other parameters constant, data on the drug’s area and tailing factor were collected and presented in Tables 3 and 4. The results indicate that the method’s reliability and reproducibility were not significantly affected by these small variations, demonstrating the robustness of the method.

Overall, the validated HPTLC-densitometric method provides accurate and precise quantification of teriflunomide and its impurities in pharmaceutical samples, and it exhibits robustness in the face of minor changes in the experimental conditions.

### Characterization of degradation product of teriflunamide

The stability-indicating capability was examined by accelerated stress testing.

#### Acid degradation

**Fig. 4:**
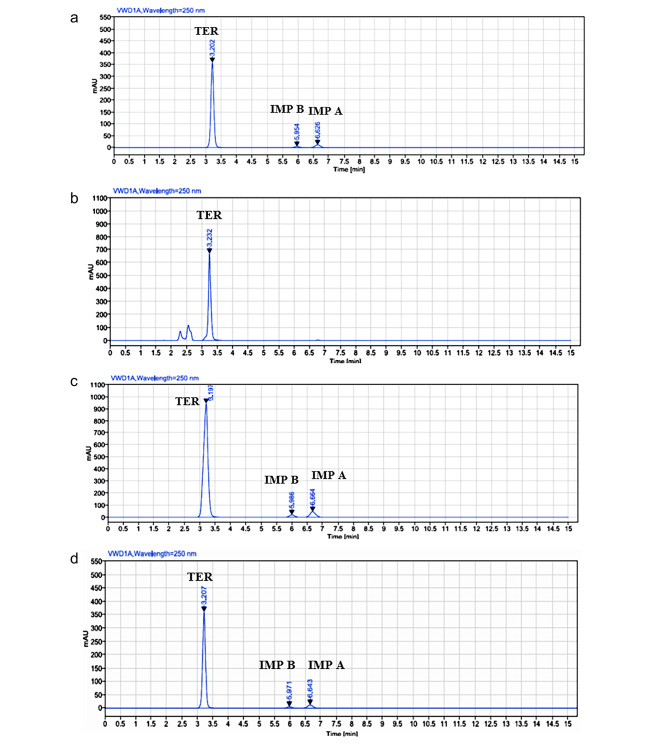
**(a)** RP-HPLC chromatogram of TER after acid degradation. **(b)** RP-HPLC chromatogram of TER after base degradation. **(c)** RP-HPLC chromatogram of TER after oxidative degradation. **(d)** RP-HPLC chromatogram of TER after thermal degradation

**Fig. 5:**
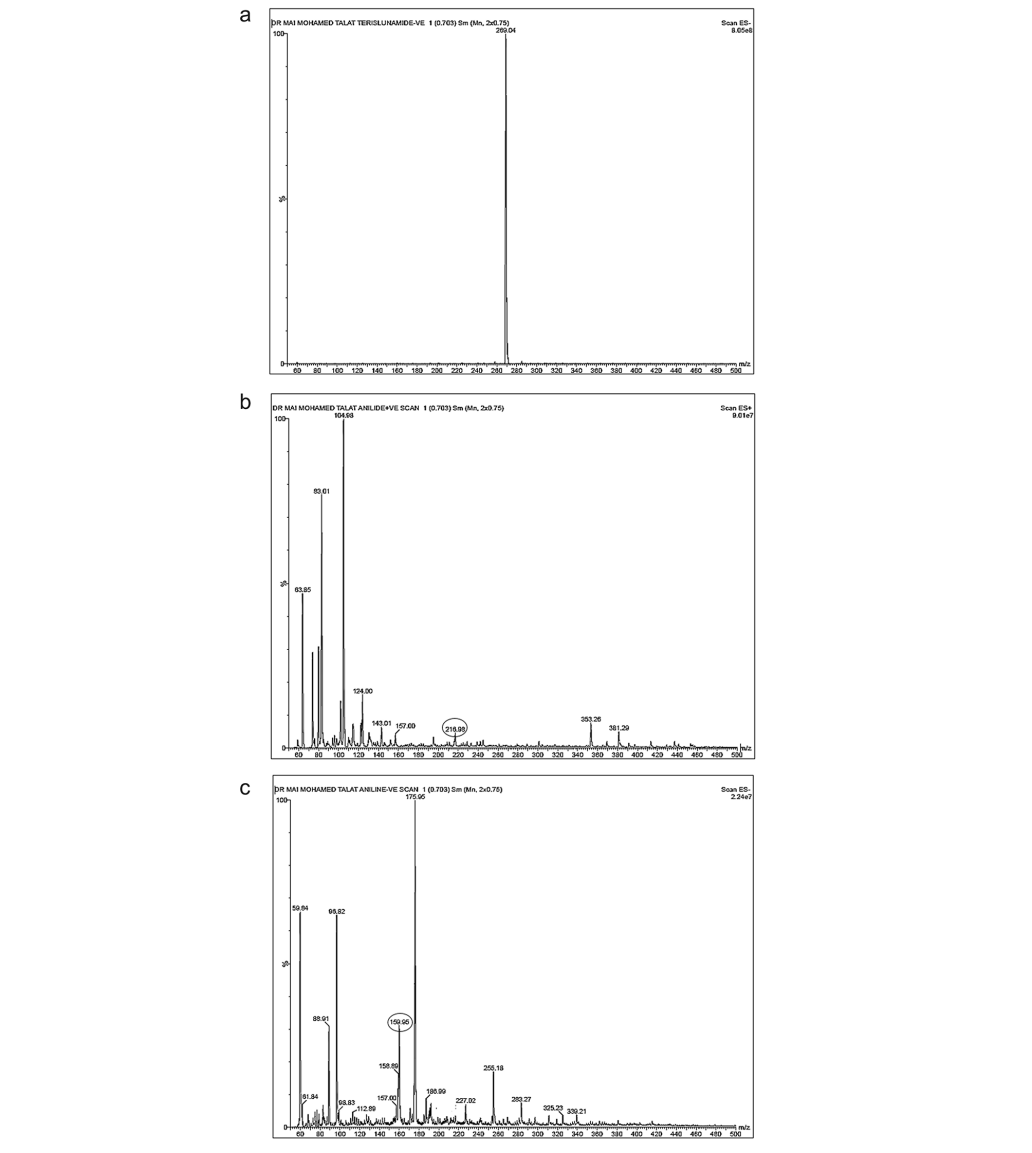
**(a)** Mass chart of intact teriflunomide (TER). **(b)** Mass chart of pure sample of 2-cyano-N-[4 (trifIuoromethyl) phenyl] acetamide (IMP B). **(c)** Mass chart of pure sample of 4-(trifluoromethyl) aniline (IMP A)

**Fig. 6:**
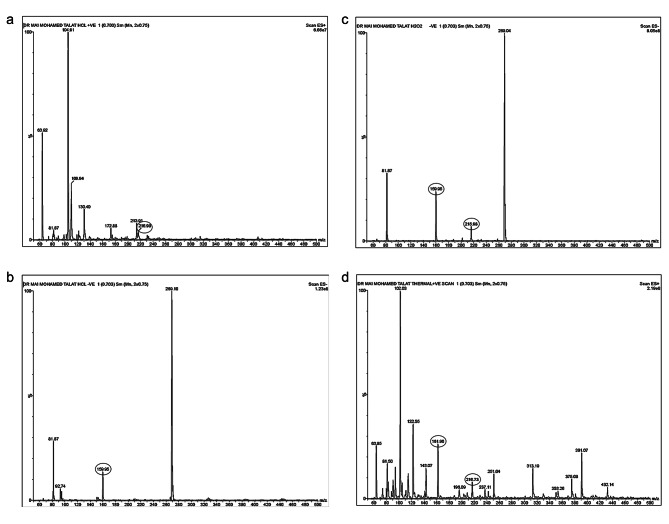
**(a)** Mass chart for acid degradation products of TER in +ve mode. **(b)** Mass chart for acid degradation products of TER in −ve mode. **(c)** Mass chart of oxidative degradation products of TER. **(d)** Mass chart of thermal degradation products of TER

Teriflunomide was found to be relatively stable under acid conditions, undergoing partial degradation to form both 4-(trifluoromethyl) aniline (IMPA) and a very small amount of 2-cyano-N-[4-(trifluoromethyl)phenyl] acetamide (IMPB), as shown in Fig. (4a). This degradation pathway was confirmed by comparing the mass spectrometry (MS) spectra of the intact drug (m/z 269) (Fig. 5a) with those of pure samples of IMPB (Fig. 5b) and IMPA (Fig. 5c). The MS spectra of the acid degradation products were obtained in both positive and negative ion modes (Fig. 6a and b).

In the positive ion mode, IMPB appeared as a fragment with an m/z of 216, resulting from the removal of a carbon atom from the cyanogen group. In the negative ion mode, IMPA appeared with an m/z of 160. These mass spectra provide evidence of the formation of degradation products during acid degradation of teriflunomide.

These findings highlight the susceptibility of teriflunomide to acid-induced degradation, leading to the formation of IMPA and IMPB. The characterization of these degradation products using mass spectrometry aids in understanding the degradation pathways and assists in the development of stability-indicating methods for teriflunomide analysis.

#### Base degradation

When teriflunomide was subjected to degradation under alkaline conditions, it demonstrated higher resistance compared to acid degradation. As a result, the chromatogram of intact teriflunomide under alkaline conditions (Fig. 4b) showed minimal degradation. This indicates that teriflunomide is relatively stable under alkaline conditions and maintains its integrity without significant degradation. This finding further supports the stability of teriflunomide under different environmental conditions and highlights its potential as a stable pharmaceutical compound.

#### Oxidative degradation

Under oxidative conditions, teriflunomide undergoes slight degradation, resulting in the formation of both 4-(trifluoromethyl) aniline (IMPA) and 2-cyano-N-[4-(trifluoromethyl) phenyl] acetamide (IMPB), as shown in Fig. (4c). The mass spectrum of the oxidative degradation products confirms the presence of IMPB with an m/z of 216 and IMPA with an m/z of 160, as depicted in Fig. (6c). This indicates that teriflunomide is susceptible to oxidative degradation, which leads to the formation of these degradation products. Monitoring and understanding the degradation pathways of teriflunomide under different conditions are essential for ensuring its stability and efficacy in pharmaceutical formulations.

#### Thermal degradation

Under thermal conditions, teriflunomide undergoes incomplete degradation, resulting in the formation of both 4-(trifluoromethyl) aniline (IMPA) and 2-cyano-N-[4-(trifluoromethyl) phenyl] acetamide (IMPB), as shown in Fig. (4d). The mass spectrometry analysis of the thermal degradation products confirms the presence of IMPB with an m/z of 216 and IMPA with an m/z of 160, as depicted in Fig. (6d). This indicates that thermal stress can lead to the degradation of teriflunomide, resulting in the formation of these degradation products. Monitoring the thermal stability of teriflunomide is crucial for ensuring its quality and efficacy during storage and use.

### Method validation

#### ***Linearity and range***

Calibration curves, which illustrate the linear association between peak area and subsequent concentration, were generated for both methods. The slopes, intercepts, correlation coefficients, and ranges are presented in Table 2.

#### ***Accuracy***

In Table 2, one can see that HPLC and HPTLC achieved % recoveries of 100.51 ± 0.41% and 101.01 ± 0.32%, respectively, which indicates the high accuracy of the suggested procedures.

#### ***Precision***

Precision is assessed through both repeatability and intermediate precision. In Table 2, you can find the calculated relative standard deviations (RSD %) for both methods, which were 0.751 and 1.252 for repeatability, and 1.245 and 1.855 for intermediate precision, for HPLC and HPTLC, respectively.

#### ***Detection and quantitation limits***

Forced degradation, involving hydrolysis, oxidation, and thermal stress. This process generated mixtures containing the target drugs and their impurities, which were subsequently analysed using the developed methods. The results, which demonstrate the methods’ reliability, are presented in Table 2, revealing a limit of detection (LOD) of 1 µg/mL and 0.5 µg/band, as well as a limit of quantification (LOQ) of 3 µg/mL and 1 µg/band for HPLC and HPTLC, respectively.

#### ***Robustness***

The robustness of the proposed methods is assessed by introducing minor experimental variations during the analytical procedure. The robustness of the methods is quantified using the relative standard deviation (RSD%), resulting in pooled values of 0.980 for HPLC and 1.606 for HPTLC, as depicted in Table 3.

### Application to pharmaceutical formulations

The successful application of the recommended chromatographic methods for the determination of TER in Aubagio® tablets is a positive outcome. The standard addition approach was used to validate the proposed procedures, and the results, as shown in Table 4, demonstrated good agreement with the labelled levels of TER in the tablets, indicating the accuracy of the methods. Importantly, no interference from excipients was observed, further confirming the specificity of the methods.

To compare the accuracy of the proposed method with a reported method in drug substance, the F-test and Student t-test were utilized [18, 19]. The calculated F- and t-values in Table 5 were found to be less than the critical values. This indicates that, at a 95% confidence level, there is no significant difference between the accuracy and precision of the proposed method and the reported method. This further validates the reliability and suitability of the proposed chromatographic methods for the determination of TER.

**Table 5:** Statistical comparison between the results obtained by the proposed HPLC &HPTLC methods and the reported method for analysis of TER in its pure powdered form

Parameters	Proposed Methods		Reported method	
	HPLC	HPTLC	HPLC(19)*	HPTLC(18)**
**Mean Recovery**	100.02%±0.39	99.50%±0.74	99.81%±0.47	99.11%±0.80
***n***	9	9	9	9
**SD**	0.39	0.74	0.47	0.80
**Variance**	0.156	0.553	0.225	0.654
**Student t-test**	1.08(2.12)	1.09(2.12)		
**F-Test**	1.44(2.44)	1.18(2.44)		

* RP-HPLC using C18, end-capped (250 × 4.6) mm, 5 μm, at 250 nm. The mobile phase A was buffer pH 3.0; while, the mobile phase B was acetonitrile (ACN) (100%) in gradient mode flow rate 1 ml/min

**HPTLC-densitometric method was done on aluminium plates coated with silica gel 60 F254 utilizing toluene: ethyl acetate: glacial acetic acid (7.5:2: 0.5 v/v/v) as the mobile phase at UV 284 nm

Overall, the successful application of the methods in the analysis of Aubagio® tablets, along with the validation and comparison results, support the robustness and accuracy of the proposed chromatographic procedures for the quantification of TER.

## Conclusion

The establishment of new stability-indicating RP-HPLC and HPTLC approaches for the determination of teriflunomide in bulk and pharmaceutical dosage forms is a significant achievement. These developed methods have been thoroughly evaluated and found to possess high sensitivity, precision, and accuracy, making them suitable for the separation and quantification of teriflunomide in routine analyses. Importantly, these methods can effectively handle the presence of impurities such as 4-(trifluoromethyl) aniline and 2-cyano-N-(4-trifluoromethylphenyl) acetamide. In addition to the RP-HPLC and HPTLC techniques, the LC/MS method was employed to elucidate the degradation products of teriflunomide. This complementary approach provided valuable insights into the degradation pathways and helped in the identification and characterization of the degradation products. Overall, the developed stability-indicating RP-HPLC, HPTLC, and LC/MS methods offer reliable and robust tools for the analysis of teriflunomide in various forms. These methods can be effectively utilized in quality control laboratories to ensure the accurate determination of teriflunomide and to assess its stability in bulk materials and pharmaceutical dosage forms.

## Data Availability

Data will be made available on request.
